# Self-Efficacy Proxy Predicts Physical Frailty Incidence Over 8 Years in Non-Institutionalized Older Adults

**DOI:** 10.1093/geroni/igaa057.1263

**Published:** 2020-12-16

**Authors:** Melissa Hladek, Jiafeng Zhu, Brian Buta, Sarah Szanton, Karen Bandeen-Roche, Jeremy Walston, Qian-Li Xue

**Affiliations:** 1 Johns Hopkins University, Baltimore, Maryland, United States; 2 School of Public Health, Johns Hopkins University, Baltimore, Maryland, United States; 3 Johns Hopkins Bloomberg School of Public Health, Baltimore, Maryland, United States; 4 Johns Hopkins School of Medicine, Baltimore, Maryland, United States

## Abstract

Physical frailty is defined as a syndrome of decreased physiologic reserve conferring vulnerability to functional decline, mortality and other adverse outcomes in response to a stressor. One potential modifiable risk factor of frailty is self-efficacy, which is confidence in one’s ability to perform well at a task or domain in life. Self-efficacy is associated with improved health behavior and decreased chronic disease burden but has not been studied extensively in frailty research. Therefore, the purpose of this study was to evaluate a general self-efficacy proxy measure’s ability to predict frailty in a nationally representative sample of older adults using data from the National Health and Aging Trends Study (NHATS) collected from 2011-2018. 4,835 older adults (65+) were dichotomized into low and high self-efficacy groups using the one-item self-efficacy proxy measure in NHATS. The Physical Frailty Phenotype was used to assess frailty. A discrete time hazard model was used to obtain incident hazard ratios of frailty in two models. Model 1 was adjusted for age, race, sex, education and income. Model 2 contained Model 1 covariates and activities of daily living and co-morbidities. We found that low self-efficacy predicted a 41% increased risk of developing frailty over 8 years after adjustment for socio-demographics (P<0.0001) and a 27% risk of incident frailty after further adjustment for activities of daily living and co-morbidities (P=0.004). This study provides preliminary evidence that self-efficacy may be a key modifiable element to incorporate into multi-modal frailty interventions.

